# Clinical retrospective analysis of 14 cases of superficial siderosis of the central nervous system

**DOI:** 10.1186/s12883-026-04812-w

**Published:** 2026-03-17

**Authors:** Ying Ji, Hafiz Khuram Raza, Bing Xu, Hailun Wang, Yu Kong, Xiaowen Li, Jin Tian, Xiaosa Sun, Gang Liu, Hao Chen, Keke Li

**Affiliations:** 1 Department of Neurology, the affiliated Hospital of Xuzhou Medical University, Xuzhou, Jiangsu 221002 China; 2 Department of Neurology, the affiliated Suzhou Hospital of Nanjing Medical University, Suzhou, Jiangsu 215000 China; 3 Department of Imaging, the affiliated Hospital of Xuzhou Medical University, Xuzhou, Jiangsu 221002 China; 4 Academician Expert Workstation of Fengxian District, Shanghai Yuansong Biotechnology Limited Company, Shanghai, 201400 China; 5 Department of Otolaryngology, the affiliated Hospital of Xuzhou Medical University, Xuzhou, Jiangsu 221002 China; 6 Department of Pathology, Guangzhou KingMed Clinical Diagnostics Center, Guangzhou, 510330 China; 7 Department of Neurology, Xuanwu Hospital Capital Medical University, Beijing, Beijing, 100053 China

**Keywords:** Central nervous system, Surface iron deposition disease, Progressive hearing loss, Ataxia, Cerebrospinal fluid, Magnetic resonance imaging

## Abstract

**Background:**

Superficial siderosis of the central nervous system (SSCNS) is a rare neurodegenerative disorder resulting from chronic hemorrhage into the subarachnoid space. This study aims to summarize its clinical and paraclinical features to improve clinical recognition.

**Methods:**

We retrospectively analyzed the medical records of 14 patients diagnosed with SSCNS at the Affiliated Hospital of Xuzhou Medical University and Xuanwu Hospital between August 2011 and April 2023. Data on clinical manifestations, cerebrospinal fluid (CSF), imaging findings, treatment, and prognosis were collected and summarized.

**Results:**

Among 14 patients (6 males, 8 females; mean age 53.1 years), the most common clinical manifestations were hearing loss (14/14, 100%), ataxia (12/14, 85.7%), and pyramidal tract dysfunction (12/14, 85.7%). Brain MRI revealed characteristic superficial siderosis in all cases, with susceptibility-weighted imaging (SWI) demonstrating superior sensitivity. Spinal cord involvement was observed in 13 patients (92.9%). CSF pressure predominantly falling within the normal range, while lumbar CSF exhibited signs of hemorrhage or yellowing, elevated red blood cell counts, and varying degrees of increased protein content. Sensorineural hearing loss was confirmed in 7/8 tested patients (87.5%). Potential bleeding sources were identified in 12 patients (85.7%), including prior surgery, trauma, and vascular abnormalities. Treatment was primarily symptomatic, with most patients (12/14) showing minimal improvement in core symptoms during hospitalization.

**Conclusion:**

Superficial siderosis of the central nervous system is a progressive neurodegenerative disorder characterized by the clinical triad of hearing loss, ataxia, and pyramidal tract dysfunction. MRI, particularly T2-weighted imaging (T2WI) and SWI sequences demonstrating a characteristic peripheral low-signal band, provides high diagnostic sensitivity and represents the cornerstone of diagnosis. Early identification remains challenging yet critical, while management should focus on locating the underlying bleeding source and providing comprehensive supportive care.

**Trial registration:**

Not applicable.

## Introduction

Superficial Siderosis of the Central Nervous System (SSCNS) is a rare neurological degenerative disorder resulting from chronic and recurrent subarachnoid hemorrhages, leading to the deposition of hemosiderin on the delicate meninges and the adjacent cerebral parenchymal surface [1]. This process inflicts lasting and irreversible damage to the nervous system. The triad of cardinal symptoms includes chronic and progressive hearing loss, cerebellar ataxia, and myelopathy, often accompanied by various manifestations of cranial nerve and cerebrospinal cord involvement [1–3]. On histopathological analysis, the brainstem, cerebellum, and spinal cord typically display hemosiderin, iron, and ferritin deposits alongside secondary nerve cell degeneration, glial hyperplasia, and demyelination changes. The presumed cause is an ongoing, subtle, and recurrent leakage of minute quantities of red blood cells (RBCs) into the subarachnoid space [4].

While the initial documentation of SSCNS dates back 118 years [5], it was not until 2007 that the first diagnosed case emerged in China [6], primarily due to the rarity of clinical presentations and the historical reliance on postmortem pathological diagnoses. The clinical manifestations of SSCNS are exceedingly atypical, frequently leading to misdiagnoses and missed diagnoses. To address this, we examined 14 classic SSCNS cases admitted to the Affiliated Hospital of Xuzhou Medical University and Xuanwu Hospital of Capital Medical University. This study aimed to consolidate our understanding of the disease by summarizing the clinical presentations and results from imaging, lumbar punctures, and audiological examinations. Additionally, we engaged with existing literature to explore potential etiologies, pathogenesis, and early identification markers for SSCNS, thus enhancing clinicians’ knowledge of this condition.

## Materials and methods

### Research subjects

Based on the diagnosis of “Superficial Siderosis of the Central Nervous System” as the definitive conclusion, we conducted a comprehensive review of hospitalization case data obtained from the Affiliated Hospital of Xuzhou Medical University and Xuanwu Hospital of Capital Medical University. A total of 14 cases whose discharge diagnoses met the criteria for SSCNS were identified and the hospitalization period ranged from August 2011 to April 2023.

The diagnostic criteria utilized were as follows: Patients presented with clinical complaints of walking instability or ataxia, and diagnostic imaging, including T2 images or other iron-sensitive sequences, revealed the presence of low-signal rings on the surface of the brain stem, cerebellum, and spinal cord, thereby confirming the diagnosis of SSCNS.

### Research methods

This retrospective study analyzed the clinical data of 14 patients, encompassing demographic characteristics, clinical presentations, temporal sequence of symptom onset, laboratory and imaging findings, administered treatments, and subsequent prognosis. However, the inherent limitations of a retrospective design should be acknowledged, including potential selection bias, information bias due to incomplete or inconsistent medical records, the inability to control for confounding factors, and loss to follow-up.

## Results

### Clinical data

The study cohort comprised 14 SSCNS patients with a mean age of 53.1 years (range 35–68), consisting of 6 males (42.9%) and 8 females (57.1%). The disease duration varied from 1 to 20 years. 

#### Clinical manifestations

The clinical presentation of SSCNS in our cohort demonstrated considerable heterogeneity while revealing consistent core features. The classic diagnostic triad was notably present: progressive hearing loss was universally observed (14/14, 100.0%), while ataxia and pyramidal tract dysfunction were each identified in 12 patients (12/14, 85.7%). All three classic symptoms were present concurrently in 10 of the 14 cases (71.4%). Vestibular symptoms, primarily dizziness, affected 11 cases (11/14, 78.6%). Additional frequent manifestations included dysarthria (5/14, 35.7%), sphincter disturbances (5/14, 35.7%), and memory impairment (4/14, 28.6%). Less common findings included hypoesthesia (2/14, 14.3%) and isolated symptoms such as cough, hoarseness, episodic unconsciousness, headache, tremor, and paroxysmal vertigo, each occurring in single cases (1/14, 7.1%).

#### Initial symptoms

Analysis of presenting symptoms revealed dizziness as the most common initial manifestation (7/14, 50.0%), followed by hearing loss (3/14, 21.4%) and ataxia (3/14, 21.4%). The remaining case (1/14, 7.1%) presented with predominant pyramidal signs characterized by bilateral lower limb weakness (left-sided predominance), accompanied by persistent numbness and discomfort below the T4 dermatome. This presentation was associated with complete loss of pain and temperature sensation below the nipple plane, consistent with a suspended sensory level at T4.

#### Neurological signs

Comprehensive neurological examination revealed objective signs in 12 patients (12/14, 85.7%). Pyramidal tract involvement was universally documented in these patients (12/12, 100.0%), manifesting as unilateral or bilateral hyperreflexia, extensor plantar responses, and spasticity. Truncal ataxia was consistently observed in all examined patients (12/12, 100.0%). Ocular motor abnormalities, specifically ocular dysmetria, were present in 3 cases (3/12, 25.0%). Sensory examination demonstrated dissociated sensory loss with preserved vibration sense in one patient (1/12, 8.3%) who exhibited decreased pinprick sensation below the T8 dermatome, and another patient (1/12, 8.3%) showed superficial hypoesthesia below the T12 level with intact vibration perception.

#### History of past illness

Review of medical histories identified several potential predisposing factors for SSCNS. One patient (1/14, 7.1%) had undergone previous pituitary tumor resection. Another patient (1/14, 7.1%) received extensive orthopedic physiotherapy for cervical and lumbar spondylosis two years prior to symptom onset. Additional significant histories included: remote vertebral trauma (1/14, 7.1%), documented subarachnoid hemorrhage (1/14, 7.1%), and cerebral hydatid infection (1/14, 7.1%). Minor traumatic events were documented in two cases (2/14, 14.3%), including one patient with head trauma from impacting a door frame and another with incidentally discovered thoracic compression fracture, both suggesting possible associations with minor traumatic etiologies.

The specific clinical data of the 14 patients are shown in Table 1. and Table 2.

**Table 1:** Summary of demographic and clinical characteristics in 14 SSCNS patients

Variable	Statistical Description
Female, n (%)	8(57.1%)
Male, n (%)	6(42.9%)
Age(years)	53.1 ± 10.1
Disease Duration(years)	3.0(1.4, 4.0)
*Cinical manifestation*	
Dizziness, n (%)	11(78.6%)
Ataxia, n (%)	12(85.7%)
Hearing loss, n (%)	14(100.0%)
Pyramidal tract dysfunction, n (%)	12(85.7%)
Nystagmus, n (%)	3(21.4%)
Tinnitus, n (%)	3(21.4%)
Dysphagia, n (%)	3(21.4%)
Dysarthrosis, n (%)	5(35.7%)
Memory deterioration, n (%)	4(28.6%)
Abnormal urine and feces, n (%)	5(35.7%)
Abnormal sensation, n (%)	2(14.3%)

**Table 2:** Clinical manifestations and auxiliary examinations of the 14 patients with SSCNS

Variable	1	2	3	4	5	6	7	8	9	10	11	12	13	14
Disease course (year)	2	2	1	1.5	4	3	3	3	1	3	5	20	4	1
*Cinical manifestation*														
Dizziness	-	+*	-	+*	-	+*	+	+*	+*	+*	+	+	+	+*
Ataxia	+	+	-	+	+*	+	+	-	+	+	+	+*	+*	+
Hearing loss	+*	+	+	+	+	+	+*	+	+	+	+*	+	+	+
Pyramidal tract dysfunction	-	+	+*	+	+	+	+	+	+	-	+	+	+	+
Nystagmus	-	-	-	-	+	-	-	-	-	-	+	-	+	-
Tinnitus	-	+	-	-	-	-	+	-	-	+	-	-	-	-
Dysphagia	-	-	-	+	-	+	-	-	-	-	-	-	+	-
Dysarthrosis	-	-	-	-	+	+	-	+	-	-	-	+	+	-
Memory deterioration	-	-	-	-	+	-	-	+	+	-	+	-	-	-
Abnormal urine and feces	-	+	+	-	-	-	-	+	-	+	+	-	-	-
Abnormal sensation	-	+	+	-	-	-	-	-	-	-	-	-	-	-
*Accomplish MRI*														
Head	+	+	+	+	+	+	+	+	+	+	+	+	+	+
Neck	+	+	+	+	+	+	+	+	+	+	+	+	/	+
Chest	/	+	+	/	+	+	+	/	+	+	+	/	/	+
Waist	+	+	/	/	/	/	/	/	+	+	/	/	/	+
*Accomplish MRM*	/	+	/	/	/	/	/	/	/	/	/	/	/	/
*MRI abnormal signal range*														
Head	+	+	-	+	+	+	+	+	+	+	+	+	+	+
Neck	+	+	+	+	+	+	+	+	+	+	+	-	-	+
Chest	-	+	+	-	+	+	+	-	+	+	+	-	-	+
Waist	+	-	-	-	-	-	-	-	+	+	-	-	-	+
*Intraspinal fluid*	-	+	-	-	-	-	-	-	-	+	-	-	-	-
*Large vessel examination*														
Head + spinal cord DSA	***/***	***/***	***/***	***/***	***/***	***/***	***/***	***/***	***/***	***/***	***/***	***/***	***/***	***/***
Head and neck CTA	/	+	/	/	+	/	/	/	+	+	+	/	+	+
MRA	/	/	+	+	/	/	+	/	/	/	/	/	+	/
*Lumbar puncture*	+	+	+	+	+	+	+	+	+	+	+	+	/	/
*Iron metabolism*	+	+	/	+	+	+	+	/	+	/	/	/	/	/
*Electric listening test*	/	+	/	/	/	+	+	+	+	+	/	+	/	+
*Vestibular function examination*	+	/	/	/	/	+	+	/	/	/	+	/	/	+

1–14 is the serial number of SSCNS patient; + means yes, -means no; * means first symptoms;/ means no or cannot be counted

### Imaging results

#### Head MR

All 14 patients (100%) demonstrated characteristic hypointense rims on brain MRI, involving the cerebellar surfaces, brainstem, and craniocervical junction. SWI and T2WI sequences provided superior visualization of these hemosiderin deposits compared to conventional T2 imaging in all cases.

#### MRI of spinal cord

Comprehensive spinal MRI evaluation demonstrated: cervical spine involvement in 12 patients (12/13, 92.3%), thoracic spine involvement in 9 patients (9/13, 69.2%), and lumbar spine involvement in 4 patients (4/13, 30.8%). Hemosiderin deposition patterns revealed: complete neuraxis involvement (cervical/thoracic/lumbar) in 3 cases (3/13, 23.1%), cervicothoracic distribution in 6 cases (6/13, 46.1%), cervicolumbar distribution in 1 case (1/13, 7.7%), and isolated cervical cord deposition in 2 cases (2/13, 15.4%). One patient (1/13, 7.7%) showed no spinal cord abnormalities. Fat-suppressed T2-weighted sequences demonstrated superior sensitivity for hemosiderin detection compared to conventional T2 sequences in selected cases.

#### Magnetic resonance myelography

**Fig. 1:**
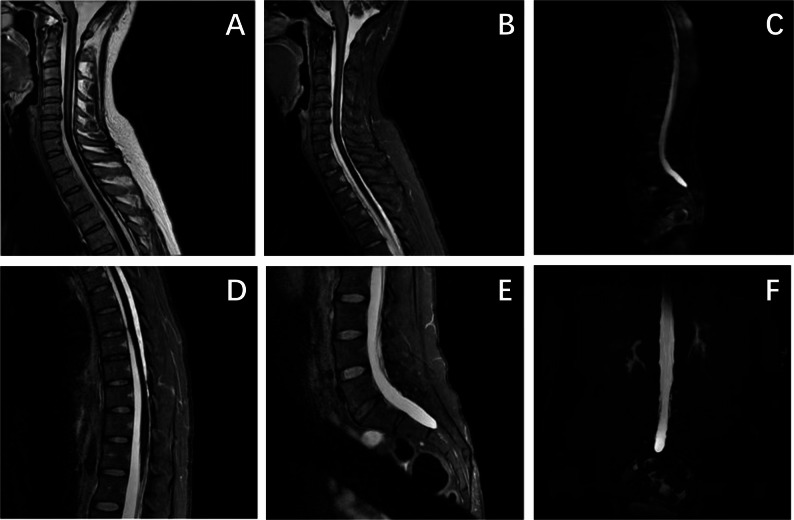
The spinal cord MRM of patient 2 A Sagittal T2, B-E-F Sagittal T2 fat sat, C-F Myelography

In one patient, magnetic resonance myelography (MRM) was conducted, revealing a gradual tapering and atrophy of the spinal cord, accompanied by a linear T2 low-signal shadow on the surface and an enlarged subarachnoid space along the spinal cord surface. The MRM showed that the cerebrospinal fluid signal in the lumbosacral spinal canal was continuous, and there was no obvious interruption or compression the nerve roots as well. This diffuse reduction in T2 signal within the spinal cord corresponded with hemosiderin deposition on the surface of the central nervous system (Fig. 1).

#### Other radiographic changes

**Fig. 2:**
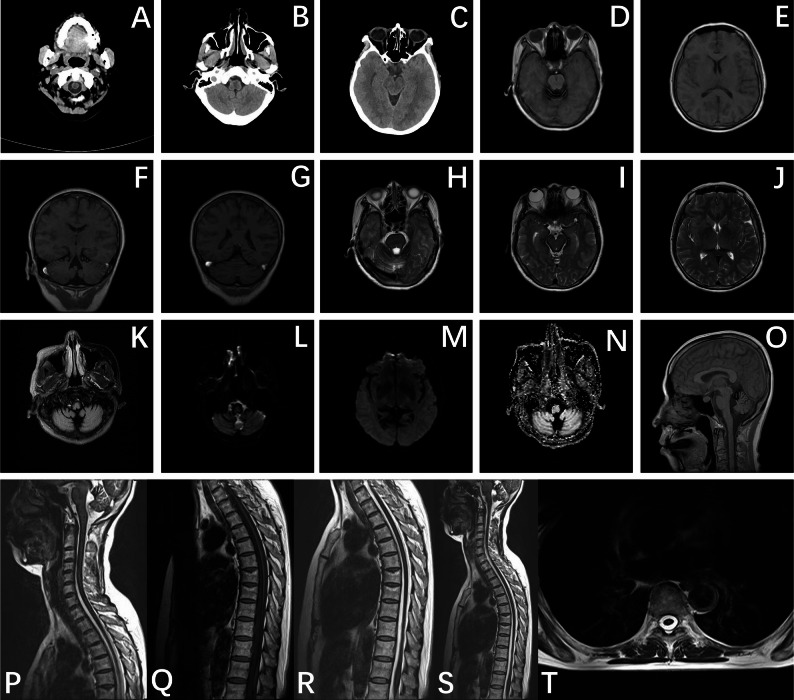
Case 5 A-C head CT: the surface of medulla oblongata, cerebellar hemispheres and around the fourth ventricle was covered with slightly higher linear signal; D, E, H-N cross-sectional head MRI, F-G coronal-section head MR: some sulcus gyrus widened and deepened, Cerebral stem and cerebellar atrophy, At the surface of the bilateral cerebral and cerebellar hemispheres sulci, cerebral split, cisterna and brainstem in the T1WI sequence, The T2WI, T2 Flair, DWI and SWI sequences are uniform linear sample with low signal shadow coverage; O-S MRI of sagittal head and cervicothoracic spine: spinal cord thinning, atrophy, enlargement of subarachnoid space, Brain stem and spinal cord surface in T1WI sequence, The T2WI sequence is a uniform line sample with low signal shadow coverage; graph T cross section thoracic vertebrae MRI T2WI: annular low signal shadow on the spinal cord surface

**Fig. 3:**
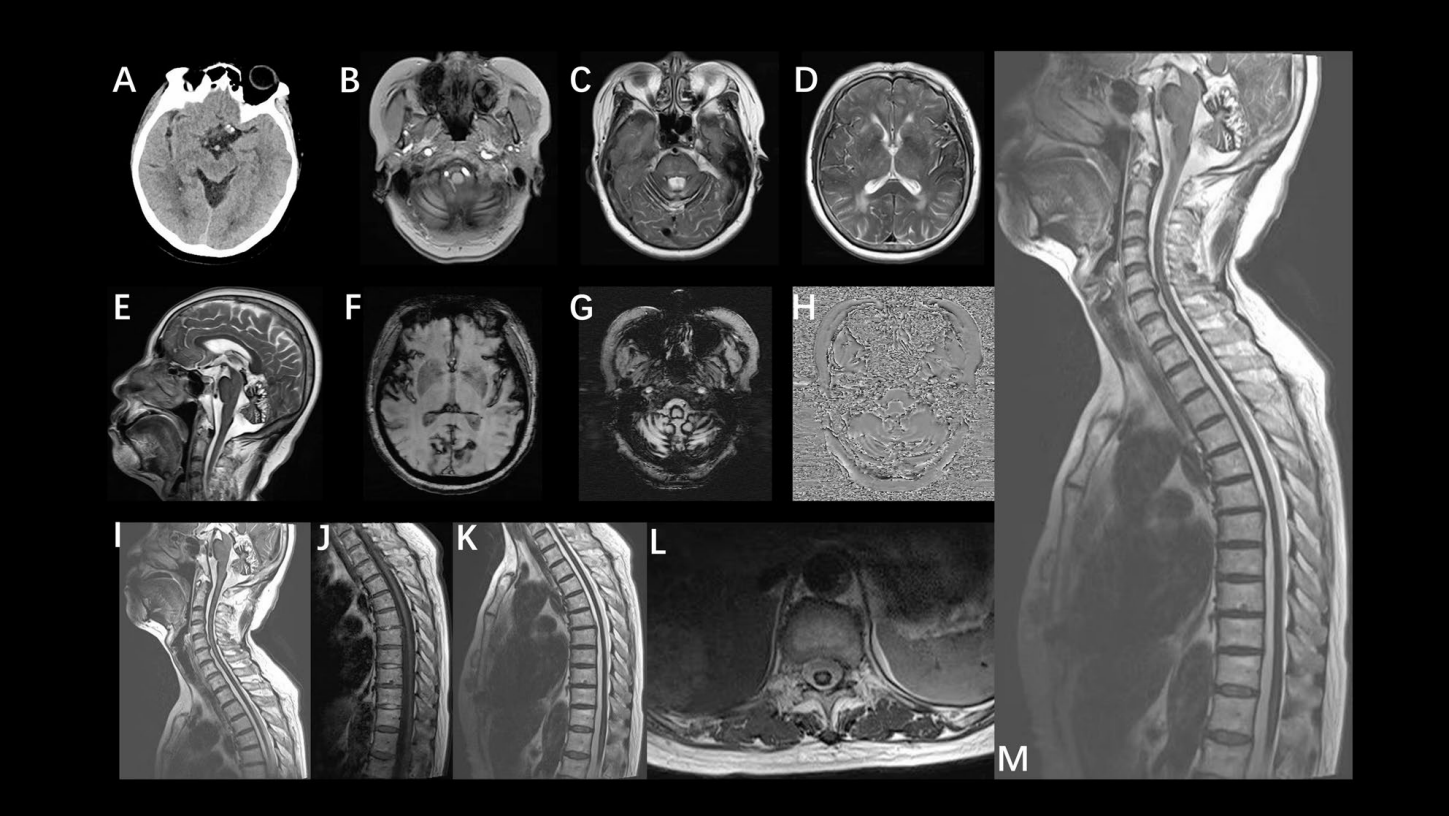
Case 6 A Head CT: the surface of midbrain, pons and along the fourth ventricle was covered with slightly higher linear signal; sagittal and axial Figure B-H head MRI: brainstem and cerebellar atrophy, the surface of brainstem, cerebellum and temporal groove circuit showed slightly higher signal in T1WI sequence, T2WI, DWI and SWI sequence showed uniform low signal coverage on the supratentorial and subtentorial structural surfaces of the cerebellum. I-M MRI of sagittal /cross head and cervicothoracic spine: abnormal signals in the cervical 3–7 and thoracic medullary cord are consistent with the manifestations of SSCNS

Cerebellar atrophy was universally observed (14/14, 100.0%), with a predilection for the vermis. Spinal cord atrophy was documented in 3 cases (3/14, 21.4%), including 2 with combined cervical and thoracic atrophy and 1 with isolated cervical cord atrophy. Brainstem atrophy was present in 1 case (1/14, 7.1%). Unique structural abnormalities included: ventral epidural effusion at T1-T8 levels (1/14, 7.1%), sacral canal occupying lesion (1/14, 7.1%), vertebral stenosis (1/14, 7.1%), and T10 compression fracture without evident dural disruption (1/14, 7.1%). Degenerative spinal changes were universally present across all cases (14/14, 100.0%) (Figs. 2 and 3).

#### Large blood vessel examination

Among 7 patients undergoing cerebral and cervical computed tomographic angiography (CTA), 2 cases (2/7, 28.6%) demonstrated normal vascular anatomy. The remaining 5 patients (5/7, 71.4%) exhibited mild to moderate arteriosclerotic changes, with specific cerebrovascular stenosis observed in 4 cases (4/7, 57.1%): posterior cerebral artery stenosis (3/7, 42.9%), vertebral artery stenosis (2/7, 28.6%), and severe left anterior cerebral artery stenosis (1/7, 14.3%). Additionally, magnetic resonance angiography (MRA) was performed in 4 of the 14 patients (4/14, 28.6%), revealing no vascular abnormalities in either the cerebral or spinal circulation.

### CSF characteristics

Cerebrospinal fluid examination was performed in 12 patients (12/14, 85.7%). Manometric pressure measurements demonstrated normal values in 10 cases (10/12, 83.3%), with one case each of elevated (230 mmH_2_O) and decreased (70 mmH_2_O) pressure. Macroscopic examination revealed hemorrhagic CSF in 4 cases (4/12, 33.3%) and xanthochromic fluid in 3 cases (3/12, 25.0%), with the remaining 5 cases (5/12, 41.7%) showing normal appearance. Cytological analysis demonstrated significantly elevated erythrocyte counts (0–10,315 × 10^6^/L) in all cases, while leukocyte counts remained within normal limits (0–32 × 10^6^/L). Biochemical analysis revealed protein elevation of varying degrees (0.29–3.01 g/L) in all 12 patients (100%), with glucose and chloride levels maintained within normal reference ranges (Table 3) .

**Table 3:** CSF characteristics of 14 patients with SSCNS

Variable	1	2	3	4	5	6	7	8	9	10	11	12	13	14
*Lumbar puncture*	+	+	+	+	+	+	+	+	+	+	+	+	/	/
Lumbar puncture pressure (mmH _2_0)	230	90	88	/	70	/	90	100	/	/	130	118	/	/
Cerebrospinal fluid appearance	hemorrhagic	hemorrhagic	hemorrhagic	transparent	transparen	transparen	transparen	xanthochromic	xanthochromic	hemorrhagic	xanthochromic	transparen	/	/
Total CSF cell count / white blood cell count (* 10 ^ 6 / L)	1204/4	10,315/15	21/14	4/4	1313/13	4/4	0	5932/32	1002/2	4806/6	2206/-	2/2	/	/
Sugar (mmol/L)	3	2.6	N	N	2.93	/	3.6	2.69	3.29	3.42	N	3.7	/	/
Chloride (mmo / L)	124	123	N	N	127	/	123	124	125	119	130	124	/	/
Cerebrospinal fluid protein (g / L)	0.81	0.79	0.52	/	0.54	/	0.6	3.01	0.29	0.415	1.677	0.4	/	/

1–14 is the serial number of SSCNS patients,; + indicates yes, N is the normal range value, and / indicates that it is not done or cannot be counted

### Hearing and vestibular function examination

Audiometric assessment in 8 patients (8/14, 57.1%) demonstrated: bilateral sensorineural hearing loss (4/8, 50.0%); asymmetric configurations including severe-to-profound right conductive hearing loss with contralateral high-frequency sensorineural deficit (1/8, 12.5%), left mixed with right sensorineural hearing loss (1/8, 12.5%), and right conductive-predominant mixed hearing loss with type B tympanogram (1/8, 12.5%); normal findings (1/8, 12.5%). Vestibular testing in 5 patients (5/14, 35.7%) uniformly revealed semicircular canal hypofunction with diminished vestibular responses (5/5, 100.0%), absent spontaneous nystagmus.

### Possible etiology analysis

A potential bleeding source was identified in 12 patients (12/14, 85.7%), while the etiology remained undetermined in 2 cases (2/14, 14.3%). Dural defects, encompassing postoperative or traumatic causes, which represented the most frequent mechanism and were implicated in 7 patients. This mechanism may permit the occurrence of chronic micro-hemorrhages. It accounted for 50.0% (7/14) of the entire cohort and 58.3% (7/12) of patients with an identified source. Other identified sources included: vascular anomalies in 3 patients (3/12, 25.0%), consisting of subarachnoid hemorrhage or cavernous malformations (2/12, 16.7%) and a familial vascular malformation syndrome indicative of genetic predisposition (1/12, 8.3%); and inflammatory/infectious lesions (cerebral echinococcosis or a tuberculous Rathke’s cleft cyst) in 2 patients (2/12, 16.7%).

### Treatment and prognosis

Therapeutic management was heterogeneous across the cohort. One patient (1/14, 7.1%) received a neuroprotective regimen consisting of idebenone, mecobalamin, coenzyme Q10, and vitamin B1. This intervention led to an improvement in dizziness at discharge but yielded only minimal benefit for hearing loss and gait instability. Another patient (1/14, 7.1%) was treated with hyperbaric oxygen therapy alongside comprehensive management aimed at blood pressure regulation, glycemic control, plaque stabilization, cerebral circulation enhancement, and neurotrophic support. Modest improvements in dysarthria and ambulation were noted, although neurological examination findings showed no significant change. The remaining 12 patients (12/14, 85.7%) received primarily symptomatic management focused on optimizing cerebral circulation, relieving spasm and pain, and providing neural nutritional support. No targeted disease-modifying therapies were administered. Notably, only one patient was available for a hospital re-evaluation after 4 years, with both clinical symptoms and imaging findings showing no significant alteration compared to baseline.

While clinical symptoms remained stable without acute worsening during the hospitalization for all patients (14/14, 100%), the overall disease course was characterized by progressive neurological deterioration and a generally unfavorable prognosis, often necessitating psychiatric co-management for associated mood disorders [7]. This pattern underscores the progressive nature of SSCNS and highlights the limited efficacy of current supportive and symptomatic treatments in halting disease progression.

Furthermore, loss to follow-up, attributable to the inherent limitations of the retrospective design such as incomplete medical records and loss of patient contact which due to changed telephone numbers, precluded a robust assessment of long-term outcomes.

## Discussion

This retrospective analysis of 14 confirmed SSCNS cases provides several clinically significant observations regarding this rare neurodegenerative disorder. Our findings both confirm established knowledge and reveal distinctive characteristics within our cohort.

### Clinical presentation and diagnostic challenges

Our study reinforces the classical clinical triad of SSCNS, with sensorineural hearing loss present in all cases (100%) [8], accompanied by high rates of cerebellar ataxia (85.7%) and pyramidal signs (85.7%). Notably, the simultaneous presence of all three classic symptoms in 71.4% of patients exceeds previously reported rates [9], suggesting this triad may be more consistent than previously recognized [1, 2]. The diagnostic challenge is compounded by the insidious disease progression, with symptom duration ranging from 1 to 20 years in our cohort. The predominance of dizziness as the initial symptom (50%) highlights the importance of considering SSCNS even when the classic triad is incomplete [10].

### Etiological spectrum and pathogenetic mechanisms

Our etiological analysis identified potential bleeding sources in 85.7% of cases, with dural defects emerging as the predominant mechanism (58.3%). The identified triggers formed a diverse spectrum including post-surgical interventions, spinal trauma, vascular abnormalities, and structural defects [2]. This diversity underscores the need for comprehensive etiological investigation in each case [2, 11]. This etiological spectrum should be further broadened to include hemorrhagic spinal neoplasms, a rare but critically important cause because they are potentially treatable [12, 13]. Spinal cord tumors, especially ependymoma and myxopapillary ependymoma, are a recognized source of chronic subarachnoid hemorrhage [13]. Recurrent micro-bleeding from their fragile vasculature can directly precipitate the pathological cascade of SSCNS. Crucially, as has been reported, the neurological symptoms of SSCNS may serve as the presenting manifestation of an occult spinal cord ependymoma, often preceding localizing signs such as back pain or radiculopathy [14]. This clinical scenario has significant therapeutic implications, as surgical resection of the causative tumor can halt disease progression [15]. Therefore, the diagnostic evaluation for SSCNS must include full neuraxial imaging (MRI of brain and whole spine) to exclude an underlying neoplasm as a potentially curable etiology. The pathogenetic process follows the established sequence of chronic subarachnoid hemorrhage, erythrocyte degradation, and hemosiderin deposition through well-characterized enzymatic pathways [10]. When deposition exceeds clearance capacity, oxidative stress and free radical formation initiate neural damage, explaining the progressive nature of SSCNS [16, 17].

### Diagnostic imaging advances

Neuroimaging remains the cornerstone of SSCNS diagnosis. Our results demonstrate the superior sensitivity of iron-sensitive sequences, with SWI and T2WI outperforming conventional T2 imaging in all cases [2, 9]. The universal presence of characteristic hypointense rims along cerebrospinal fluid-contact surfaces provides reliable diagnostic markers [10, 18]. The high prevalence of spinal cord involvement (92.3%) and cerebellar atrophy (100%) in our cohort emphasizes the importance of comprehensive neuraxis imaging.

### CSF analysis and limitations

Cerebrospinal fluid analysis demonstrated limited diagnostic specificity, though characteristic abnormalities included protein elevation of varying degrees (0.29–3.01 g/L) and frequent hemorrhagic or xanthochromic appearance. The heterogeneity in CSF findings—particularly the variable protein levels and inconsistent presence of erythrocytes—reinforces that normal CSF parameters do not exclude SSCNS [19]. Diagnosis therefore necessitates integration of clinical manifestations and definitive imaging features, such as those provided by iron-sensitive MRI sequences.

### Therapeutic implications and prognosis

The iron chelator deferiprone presents a promising therapeutic avenue for SSCNS, given its ability to cross the blood-brain barrier and evidence from observational studies suggesting it can reduce cerebral iron deposition and potentially stabilize symptoms [11, 20, 21]. However, its translation into routine clinical practice is hindered by several significant barriers. First, the lack of regulatory approval and high-level evidence presents a major constraint. Current use in SSCNS is off-label and supported predominantly by case series and observational data, rather than randomized controlled trials [11, 22–24]. This evidence gap impedes the establishment of standardized treatment protocols and inclusion in formal clinical guidelines. Second, the risk of serious adverse effects—notably neutropenia and agranulocytosis—necessitates intensive and regular hematologic monitoring [11, 24], which poses a substantial burden on healthcare systems and may limit patient access and adherence, particularly in outpatient or resource-limited settings. Third, key clinical uncertainties remain, including optimal patient selection, timing of treatment initiation, and long-term efficacy [11]. It is still unclear which patients derive the greatest benefit, whether earlier intervention prior to substantial neurological injury improves outcomes, and how durable the treatment effects are. Finally, practical challenges such as drug accessibility, cost, and the need for multidisciplinary coordination (involving neurology, hematology for monitoring, and often psychiatry) further complicate broader implementation.

Currently, disease management remains predominantly symptomatic, as reflected in our treatment outcomes. Although neuroprotective regimens and hyperbaric oxygen therapy may offer limited symptomatic relief [21], no approach has demonstrated definitive disease-modifying effects. The frequent requirement for psychological intervention highlights the considerable disease burden and underscores the importance of integrated care that addresses both neurological and psychiatric dimensions.

## Conclusions

Based on our analysis, we conclude:

1. SSCNS demonstrates consistent clinical features dominated by the classic triad of hearing loss, ataxia, and pyramidal tract dysfunction, though presentation timing and symptom combination show variability.
2. MRI with iron-sensitive sequences (particularly SWI/T2WI) provides the most reliable diagnostic method, demonstrating characteristic hemosiderin deposition patterns in all cases.
3. Comprehensive etiological investigation is essential, with dural defects representing the most common identifiable cause, followed by vascular abnormalities and spinal pathologies.
4. Current management requires multimodal symptomatic approaches combined with psychological support, while definitive treatment should address identifiable bleeding sources when possible.
5. Future research should focus on developing targeted interventions addressing iron toxicity and preventing disease progression, particularly through larger multicenter collaborations given the rarity of this condition.

Collectively, our findings complement and extend the clinical, neuroimaging, and therapeutic spectrum of SSCNS reported in previous case reports, cohort studies, and deferiprone treatment series.

### Limitations

Several methodological and practical limitations should be considered when interpreting the findings of this study. Although the dual-center retrospective design enhanced case ascertainment, the sample size remained relatively small (*n* = 14), which limits statistical power for subgroup comparisons and may affect the generalizability of our observations to all phenotypic presentations of SSCNS. The extended study period (2011–2023) introduces potential heterogeneity in imaging protocols and diagnostic criteria over time, which could influence the consistency of radiological assessments. Furthermore, variability in the application of advanced spinal and vascular imaging across participating centers may have led to under-detection of subtle structural or vascular abnormalities contributing to recurrent hemorrhage. Loss to long-term follow-up in a substantial proportion of patients—attributable to factors such as geographic mobility and incomplete contact information—precluded a robust longitudinal analysis of disease progression and treatment response. Additionally, inherent to the retrospective design are limitations including potential selection and information bias due to incomplete medical documentation, as well as the inability to systematically control for confounding variables. Future multi-center studies with standardized imaging protocols and prospective longitudinal follow-up are warranted to validate and extend the present findings.

## Data Availability

The data supporting the findings of this study are available from the corresponding author upon reasonable request.

## **Abbreviations**

SSCNS: Superficial siderosis of the central nervous system
CSF: Cerebrospinal fluid
SWI: Susceptibility-weighted imaging
T2WI: T2-weighted imaging
RBCs: Red blood cells
MRM: Magnetic resonance myelography
CTA: Computed tomographic angiography
MRA: Magnetic resonance angiography
